# Supplementary material for: Mechanical Signals Inhibit Growth of a Grafted Tumor In Vivo: Proof of Concept
Source: PLoS One. 2016 Apr 21;11(4):e0152885. doi: 10.1371/journal.pone.0152885 (PMC4839666; doi:10.1371/journal.pone.0152885)
Supplement: S1 Appendix — (PDF) [file pone.0152885.s001.pdf]

S1 Appendix: Digitalization of sections and analysis of tumor surfaces

Cell Constraint & Cancer: Proof of Concept

Alexandre Yahi

September 2013 - December 2013

Table of contents

1 Study Background 2

2 Achievements 2

2.1 Stages of the mission

2.2 Problems 2

3 Résultats 3

3.1 Correspondance of groups with those of Oncodesign 3

3.2 Documentation about the database 3

3.3 Balance sheet on the measures 3

3.4 Balance sheet on survival 4

4 Outlook 4

List of tables

1 Correspondence groups Oncodesign/Study 4

2 Assessment number of tumors measured per group 4

3 Assessment number or mouse tumors survivors per group 4

4 Cutting survival per group in number of tumors 4

Annex 5

1 Study context

The objective of this study is to achieve proof of concept of the disruptive technology for the treatment of tumors developed by the company Constraint Cell & Cancer. This mission involved a weekly active collaboration with the Curie Institute in St Cloud, mainly with Dr. Jean-Marc Guinebretière, pathologist, and regular monitoring with Dr. Remy Brossel, commissioned the study to J2S (Junior & Supelec Strategy, Junior Enterprise Supelec).

2 Achievement

2.1 Stages of the mission

Initially focused on the electromagnetic aspect of treatment, the object of the study was modified following the time against the Altran. The new goal was to focus on proof of concept according to the experiments previously outsourced to the company Oncodesign. The mission took place as follows:
- Consistent numbering tumors cuts support blades harvested by the company Oncodesign
- Scanning Philips blades on the unit at the Institut Curie St Cloud.
- Export scanned images because of a tumor image from the raw digital ones and full-scale display
- Measurement of tumors using ImageJ free software: the tumors were measured initially to determine total surface area and necrotic, then a new set of comprehensive measures was made once all exported images
- Interpretation of results Oncodesign and matching mouse / tumor
- Creating a comprehensive database in 3 tables using a clear classification on the basis of work Oncodesign and summarizing all available information on the study

2.2 Problems encountered

Several problems were encountered during the study:

- Blade digitization presented problems connecting with servers Curie Paris, slowing the progress of scans
- Using ImageJ very "manual" compared to professional software not available for study
- Nomenclature from heavy Oncodesign and distribution of very explicit groups
- Many sections unusable for digital measurements as damaged or incomplete

3 Results

3.1 Correspondence with those groups Oncodesign

The nomenclature of Oncodesign groups G1, G2 and G3 will be used only to clarify the correspondence with the groups defined in this study as well as the dependencies between large groups for statistical processing. 4 groups used in our studies are:

1. Scope + Particles (study group) (C / P)
2. Without field / With particles (Øc / P)
3. With field / Without particles (C / Øp)
4. Without field / Without particles (Øc / Øp)

Correspondence with the groups G1, G2 and G3 Oncodesign is:
Of a total of 41 mice, 82 tumors studied, the distribution per group is as follows:

3.2 Documentation on the database

The database conducted during this study consists of three tables:
- A main table "main_tumors" with the following fields: ID, group, field, particles, cot, lame1, lame2, measure.

The ID is the code of the tumor, it is the key that is found in the 3 tables. A tumor takes its unique combination of ID and cost. The group is the group as part of the study, field and particles are specified by "yes" or "no", the side with "D" or "G", the blade fields indicate the name of the blades concerning this tumor measurement is specified by "Y" or "N", depending on whether the tumor has at least one set of steps is complete or not.
- A measurement table "tumor_measurement" with fields: file_name ID, cost surface_necr, surface_viv, surface_skin, surface_muscle obs, total_surface,.

The file_name is unique and gives the name of the source image file action. The ID and cost combination refers to a single tumor, the following 5 measurements yield the surfaces in mm2 of the total tumor, the necrotic area, non necrotic area, non necrotic area side skin and non necrotic side area muscular. Finally obs indicates the status of the scanned cutting: good, med if the measurements are performed but the cut is not perfect, and not bad for a picture to be measured correctly.
- Mice_map a table with fields: ID, group_onco, days, fashion. The ID is the general identifier used in the database, group_onco refers to the group according to Oncodesign nomenclature days specifies the number of days of survival of the mouse, and mode indicates the circumstances of his death.

Side	
Left	Right
G1	
3	2
G2	
4	3
G3	
4	1

Table 1 – Correspondence groups Oncodesign/Study

3.3 Assessment on measures

On the 82 tumors studied, 38 do not have sufficient quality to perform cutting measures. Of the 44 others, the distribution of measurements is as follows (in number of tumors):

	measured	total
group 1	9	19
group 2	13	22
group 3	3	8
group 4	19	33
TOTAL	44	82

Table 2 – Assessment number of tumors measured by group

There are a total of 55 fully measured tumors, or 165 measurements without counting the preliminary steps.

3.4 Assessment on survival

The survival per group beyond the 60 days required is as follows (in number of tumors):

	survival	total
group 1	7	19
group 2	13	22
group 3	5	8
group 4	15	33
TOTAL	40	82

Table 3 – Assessment the number of mouse tumors survivors per group

With cutting by days of survival for each group:

	0-20	21-40	41-60	60+
group 1	2	9	1	7
group 2	0	9	0	13
group 3	0	3	0	5
group 4	2	15	1	15
TOTAL	4	36	2	40

Table 4 –Survival Cutting by group in number of tumors

4 Outlook

Firstly, with the correspondence between the Oncodesign groups and groups defined in this study, it is possible to re-perform statistical tests on volume taking into account the dependencies (see Annex). Furthermore, 38 tumors still impossible to measure with cuts provided: references were forwarded to JM. Guinebretière that these tumors are intersected in the context of a new series of scanning and complementary measures.

Finally, it will remain once these measures complemented the statistical part with one hand intrinsic comparison study between skin side group (West) and muscle side (East) to demonstrate the lower side muscle thickness trend (Kruskal-Wallis matched ..) as well as the comparison of the study group versus control group each and all controls.

Annex

We can note several anomalies in the numbering. First the blade has no number 3993, this tumor is not present in the samples made available and therefore not included in the study. Furthermore, the double numbering Oncodesign is dark on the marked blades 3833/4951 and 3845/4959. Finally, the 2841 tumor is in reality number 3895 on the blades, and the tumor becomes 2843 3835. There is a blur on the 4973 tumor.

Correspondence groups Oncodesign / Study by tumor number:

Side	
Left	Right
G1	
3	2
4943	
3823/2199	
4963	
3847	
4955	
4961	
3989	
4977	
G2	
4	3
2851	
4981	
3995	
3881	
3985	
3987	
3871	
3991	
4965	
4957	
4967	
2843	
4951	
4949	

Side	
Left	Right
G3	
4	1
4945	
3981	
5041	
4947	
3983	
4959	
3855	
2849	
5043	
4969	
4971	
2847	
3883	
4973	
4975	
2841	
3897	
4983	
4985	
