# Supplementary material for: Mechanical Signals Inhibit Growth of a Grafted Tumor In Vivo: Proof of Concept
Source: PLoS One. 2016 Apr 21;11(4):e0152885. doi: 10.1371/journal.pone.0152885 (PMC4839666; doi:10.1371/journal.pone.0152885)

S2 Appendix: In vivo proof of concept of effect of nanoparticles activated by a magnetic field

IN VIVO PROOF OF CONCEPT OF EFFECT OF NANOPARTICLES ACTIVATED BY A MAGNETIC FIELD ON BALB/C NUDE MICE BEARING SUBCUTANEOUS MDA-MB-231 TUMORS

STUDY PROTOCOL

STUDY NUMBER: **120005 – March 2012**

This document is confidential. It is the exclusive property of Onco*design*. No part of this document may be translated, reproduced, stored in a retrieval system, or transmitted in any form or by any means: electronic, mechanical, recording or otherwise without the prior written permission of Onco*design*.

STUDY MANAGEMENT

Testing company:

Onco*design* S.A.
20, rue Jean Mazen
B.P. 27627
F-21076 Dijon Cedex
FRANCE
☎: +33 (0)3 80 78 82 60
Fax: +33 (0)3 80 78 82 61
✉: oraguin@oncodesign.com

Study Director:

Olivier RAGUIN, Ph.D.
Date:
Signature:

Study Manager:

Marie LEBLANC
Date:
Signature:

Sponsor:

CELL CONSTRAINT CANCER
Le Mas l'hermite
331 chemin de la Poterie
13280 Raphèle-lès-Arles
FRANCE
☎:
Fax:
E-mail: remy.brossel@gmail.com

Monitor:

Rémy BROSSEL, MD.
Date:
Signature:

EXPERIMENTATION SITES

Study will be performed at:

**Onco*design* S.A.**	**BioMEP Sarl**
20, rue Jean Mazen,<br>BP 27627<br>21076 DIJON Cedex<br><br>FRANCE<br><br>☎: +33 (0)3 80 78 82 60<br>Fax: +33 (0)3 80 78 82 61	Site INRA Breteniere<br>Bâtiment A<br>21110 BRETENIERE<br><br>FRANCE<br><br>☎: +33 (0)3.80.69.31 52

TABLE OF CONTENTS

TABLE OF CONTENTS **3**
1. STUDY AIM **4**
2. MATERIALS AND METHODS **4**
2.1. Test substance **4**
2.1.1. Test substance *4*
2.1.2. Conformity certificate of test substance *4*
2.2. Test substance preparation **4**
2.3. Nanoparticle dose **4**
2.4. Route of administration **4**
2.5. Animals **4**
2.5.1. Environment *5*
2.5.2. Animal husbandry *5*
2.5.3. Food and drink *5*
2.5.4. Animal and cage identification *5*
2.5.5. Submission of animals to a magnetic field *5*
2.6. Cancer cell line and culture conditions **5**
2.6.1. Cancer cell line *5*
2.6.2. Cell culture conditions *6*
3. EXPERIMENTAL DESIGN AND TREATMENTS **6**
3.1. Efficacy study of Nanoparticles in the model of MDA-MB-231 tumor bearing Balb/c *Nude* mice 6
3.1.1. Induction of MDA-MB-231 tumors in female Balb/c Nude mice *6*
3.1.2. Treatment schedule *6*
3.1.3. Tumor samples collection *7*
3.1.4. Samples shipment *7*
3.2. Animal monitoring and termination **7**
4. DATA PRESENTATION AND MANAGEMENT **8**
4.1. Toxicity parameters **8**
4.2. Efficacy parameters **8**
4.3. Statistical tests **9**
5. APPROVAL OF THE STUDY START **9**
6. DURATION OF THE STUDY **9**
7. ARCHIVE **9**
8. CONFIDENTIAL DISCLOSURE AGREEMENT **9**
9. BIBLIOGRAPHY **9**
PROJECT TIMING **10**

1. STUDY AIM

- To study the efficacy of Nanoparticles activated by a magnetic field in the model of subcutaneous MDA-MB-231 human breast tumor bearing mice.

2. MATERIALS AND METHODS

2.1. Test substance

2.1.1. Test substance

The test substance (i.e. Nanoparticles) will be supplied by Cell Constraint Cancer.

**Substance name**	fluidMAG-D (∅ 100 nm)
**Lot number**	2202/12
**Storage condition**	+4°C
**Supplied quantity**	100 mL (100 mg/mL)

The test substance will be classified as hazardous agents and its preparation will be performed under laminar flow conditions. The personnel will be equipped with adequate protection (mask, protective gloves, and safety goggles) during test substance manipulations. All remaining test substance will be sent back to the sponsor at the end of the study.

2.1.2. Conformity certificate of test substance

The Sponsor is responsible for sending a certificate of analysis to the Study Director for each batch of test substance supplied by Cell Constraint Cancer. This certificate documents that appropriate procedures will be used to ensure that the test substance conforms to established specifications and is positively identified as the test substance.
All remaining test substance will be returned to Cell Constraint Cancer at the end of the study using a normal courier.

2.2. Test substance preparation

The vehicle for the Nanoparticles and the preparation of the Nanoparticles will be specified by the Sponsor: water

2.3. Nanoparticle dose

- The dose was specified by the Sponsor. The amount of Nanoparticles injected with cells will be 5 mg.

2.4. Route of administration

Cell Constraint Cancer chose the administration route of the Nanoparticles. Nanoparticles will be administered subcutaneously with tumor cells (at the time of tumor cells injection) to Balb/c Nude mice.

The injection volume for the Nanoparticles will be 300 µL/mouse/injection.

2.5. Animals

Forty five (45) female Balb/c *Nude* mice, 6-7 week-old and weighing 16-20 g at reception, will be obtained from Charles River (France). Animals will be observed for at least 7 days in a specific-pathogen-free (SPF) animal care unit before treatment. The animal care unit is authorized by the French ministries of Agriculture and Research (Agreement No. A21231011EA). Animal experiments will be performed according to ethical guidelines of

animal experimentation [1] and the English guidelines for welfare of animals in experimental neoplasia [2].
All procedures with animals will be submitted to the Animal Care and Use Committee of Pharmacy and Medicine University (Dijon).

2.5.1. Environment

Animals will be maintained in rooms under controlled conditions of temperature (23 ± 2°C), humidity (45 ± 10%), photoperiod (12h light/12h dark) and air exchange. Animals will be maintained in specific pathogen free (SPF) conditions and room temperature and humidity will be continuously monitored. The air handling system will be programmed for 14 air changes/hour, with no recirculation. Fresh outside air will pass through a series of filters, before being diffused evenly into each room. A positive pressure (20 ± 4 Pa) will be maintained in the experimentation room to prevent contamination or the spread of pathogens within a mouse colony. All personnel working under SPF conditions will follow specific guidelines regarding hygiene and clothing when they enter animal husbandry area (Standard Operating Procedure TEC-003/001).

2.5.2. Animal husbandry

Animals will be housed in polycarbonate cages (Tecniplast, Limonest, France) that are equipped to provide food and water. Standard area cages are used (800 cm^2), with a maximum of 10 mice (from the same treatment group) per cage. Bedding for animals will be sterile corn cob bedding (Ref LAB COB 12, SERLAB, Cergy-Pontoise, France), replaced twice a week.

2.5.3. Food and drink

Animal food will be purchased from DIETEX (Saint-Gratien, France). The type of sterile controlled granules will be irradiated RM1. Food will be provided ad libitum, being placed in the metal lid on top of the cage. Water will be also provided ad libitum from water bottles equipped with rubber stoppers and sipper tubes. Water bottles will be cleaned, filled with tap water sterilized by filtration, and replaced twice a week.

2.5.4. Animal and cage identification

The animals will be identified by two different numbers engraved on two ear tags. Each cage will be labelled with a specific code.

2.5.5. Submission of animals to a magnetic field

When submitted to the magnetic field using magnet-bearing devices, animals will be anaesthetized (with isoflurane) and body temperature of mice will be maintained within physiological levels by infrared lamps.

The magnet-bearing devices are provided by the Sponsor.

2.6. Cancer cell line and culture conditions

2.6.1. Cancer cell line

The MDA-MB-231 human cancer cell line and culture medium will be purchased and provided by **Onco***design*. The cell line that will be used is detailed in the table hereafter:

Cell line	Type	Specie	Origin
MDA-MB-231	Human breast adenocarcinoma	Human	ATCC [a]

[a] American Type Culture Collection, Manassas, Virginia, USA

- The human breast MDA-MB-231 adenocarcinoma cell line was isolated from a pleural effusion of a 51-year old Caucasian female patient with a metastatic breast adenocarcinoma [3].

2.6.2. Cell culture conditions

The MDA-MB-231 tumor cells will be grown as adherent monolayers at 37°C in a humidified atmosphere (5% CO_2, 95% air). The culture medium will be RPMI 1640 containing 2 mM L-glutamine (Ref. BE12-702F, Lonza, Verviers, Belgium) and supplemented with 10% fetal bovine serum (FBS, Ref. 3302-P282005, Lonza).

For experimental use, cells will be detached from the culture flask by a 5 to 10-minute treatment with Versene (EDTA 0.02%, Ref. # BE17-711E, Lonza), 10-fold diluted in Hanks' medium without calcium or magnesium (Ref. #BE10-543F, Lonza) and neutralized by culture medium addition. Cells will be counted in a hemocytometer and their viability will be assessed by 0.25% trypan blue exclusion assay.

Mycoplasma detection will be performed using the MycoAlert® Mycoplasma Detection Kit (Ref. #LT07-318, Lonza) in accordance with the manufacturer instructions. The MycoAlert® Assay is a selective biochemical test that exploits the activity of enzymes from mycoplasma. The viable mycoplasma are lysed and the enzymes react with the MycoAlert® substrate catalyzing the conversion of ADP to ATP. By measuring the level of ATP in a sample both before and after the addition of the MycoAlert® substrate a ratio can be obtained which is indicative of the presence or absence of mycoplasma. The mycoplasma test will be assayed in duplicate from the culture supernatants of the cell line and compared to negative and positive controls (MycoAlert® Assay Control Set, Ref LT07-518, Lonza) (Internal Standard Operating Procedure No TEC-007/002).

3. EXPERIMENTAL DESIGN AND TREATMENTS

3.1. Efficacy study of Nanoparticles in the model of MDA-MB-231 tumor bearing Balb/c *Nude* mice

3.1.1. Induction of MDA-MB-231 tumors in female Balb/c *Nude* mice

Ten millions (10^7) MDA-MB-231 tumor cells resuspended in a volume of 0.3 mL RPMI 1640 medium containing 5 mg of nanoparticles or no nanoparticles will be subcutaneously inoculated in the flanks of 45 female SWISS *Nude* mice, irradiated 24-72 hours before with a γ-source (whole body irradiation, 2 Gy, ^{60}Co, BioMEP Sarl, Bretenière, France).

Tumors cells without the nanoparticles will be injected in the left flank of all 45 mice.

Tumors cells with the nanoparticles will be injected in the right flank of all 45 mice.

The day of tumor cells injection will be considered as the day 0 (D0).

3.1.2. Treatment schedule

When the mean tumor volume reaches approximately 100-200 mm^3, 36 tumor-bearing female Balb/c *Nude* mice will be randomized into 3 groups (one group of 8 mice and two groups of 14 mice) according to their individual tumor volumes. The mean tumor volume of each group will not be different from the others (analysis of variance). The treatment schedule was chosen by Cell Constraint Cancer as follows:

- Group 1: The tumor on the left flank (containing no nanoparticles) of the eight (8) mice will be submitted to the magnetic field once a day for two consecutive hours for 21 consecutive days.
- Group 2: The tumors on the left flank (containing no nanoparticles) and on the right flank (containing nanoparticles) of the fourteen (14) mice will not be submitted to the magnetic field.

- Group 3: The tumor on the right flank (containing nanoparticles) of the fourteen (14) mice will be submitted to the magnetic field once a day for two consecutive hours for 21 consecutive days.

Treatments (that is submission to the magnetic field) will start on the day after randomization.

The table below summarizes the treatment schedule:

Group	No. mice	Nanoparticles		Submission to magnetic field		
		Left tumor	Right tumor	Left tumor	Right tumor	Duration
1	8	No	Yes	Yes	No	2 hours, every day, 21 days
2	14	No	Yes	No	No	-
3	14	No	Yes	No	Yes	2 hours, every day, 21 days

Mice will be monitored as described in section 3.2.

3.1.3. Tumor samples collection

At two timepoints during the course of the study (to be determined in agreement with the Sponsor) and at the time of final termination, three mice from groups 2 and 3 will be terminated.

Tumors will be resected and cut into two pieces. The two pieces will be fixed in 10% neutral buffered formalin.

3.1.4. Samples shipment

Tumor samples will be shipped (at ambient temperature, while in formalin) using CryoExpress courier. The shipping address will be specified by the Sponsor.

3.2. Animal monitoring and termination

All logistical issues of the study (collection, measurements, raw data, lethality, behaviour, treatment and results of autopsy will be managed using Vivo manager® software (Biosystemes, Dijon, France). The viability and behavior will be recorded every day. Body weights will be recorded twice a week.
The length and width of the tumors will be measured twice a week with calipers and the tumor volume will be estimated by the formula [4].

$$Tumor\ volume = \frac{1}{2} \times length \times width^2$$

Isoflurane (Belamont, United Kingdom) will be used to anaesthetize the animals during subcutaneous tumor cell inoculation and at termination (by cervical dislocation or cardiac puncture). During the course of the experiment, animals will be terminated if any of the following signs occurs:

- signs of suffering (*cachexia*, weakening).
- compound toxicity (hunching, convulsions).
- tumor ulcerating and remaining open.
- position of tumor interfering with movement/feeding.
- 15% body weight loss for 3 consecutive days or 20% body weight loss for 1 day.

Xenografted mice will be terminated when the tumor volume reaches a maximum volume of 2,000 mm^3.

An autopsy (macroscopic examination of heart, lungs, liver, spleen, kidneys and gastro-intestinal tract) will be performed on all terminated (scheduled) mice in the study, and, if feasible, on all moribund/found dead mice. Autopsy observations will be recorded.

4. DATA PRESENTATION AND MANAGEMENT

The raw data will be provided to the study sponsor in an Excel file format.

4.1. Toxicity parameters

The following evaluation criteria of toxicity parameters will be determined using Vivo manager® software (Biosystemes, Dijon, FRANCE) and were chosen in consultation with the sponsor.

- **Individual and mean body weights** of mice will be provided.
- **Mean body weight change (MBWC)**: Average weight change of treated animals in grams (weight day 2 minus weight day 1) will be calculated. The intervals, over which MBWC will be calculated, will be chosen as a function of body weight curves and the days of body weight measurement.

4.2. Efficacy parameters

The treatment efficacy will be assessed in terms of the effects of the test substance on the tumor volumes of treated mice relative to untreated mice.
The following evaluation criteria of antitumor efficacy will be determined using Vivo manager® software (Biosystemes, Dijon, FRANCE) and were chosen in consultation with the sponsor.

- **Individual and mean tumor volumes** will be provided.
- **Tumor doubling time** (DT) will be calculated.
- **Tumor growth inhibition** (T/C %) defined as the *ratio* of the median tumor volumes of treated *versus* untreated groups will be calculated :

$$T/C\% = \frac{\textit{Median tumor volume of treated group at DX}}{\textit{Median tumor volume of untreated group at DX}} \times 100$$

The optimal value is the minimal T/C % *ratio* reflecting the maximal tumor growth inhibition achieved. The effective criteria for the T/C % *ratio* according to NCI standards, is ≤ 42% (5).

- **Relative tumor volume** (RTV) will be calculated as follows:

$$\text{RTV} = \frac{\text{TV at } D_X}{\text{TV at } D_R}$$

TV : Tumor volume
D_X: Day of measurement,
D_R: Day of randomization.

- **Volume V and Time to reach V** will be calculated. Volume V is defined as a target volume deduced from experimental data and chosen in exponential phase of tumor growth. For each tumor, the closest tumor volume to the target volume V will be selected in tumor volume measurements. The value of this volume V and the time for the tumor to reach this volume will be recorded. For each group, the mean of the tumor volumes V and the mean of the times to reach this volume will be calculated.

4.3. Statistical tests

All statistical analyses will be performed using Vivo manager® software (Biosystemes, Dijon, FRANCE). Statistical analyses of mean body weight changes, mean tumor volumes at randomization, mean target tumor volumes V, mean times to reach the target volume V and mean tumor doubling times will be performed using the Bonferroni/Dunn test. All groups will be compared with each other. Unless otherwise stated, a p value < 0.05 will be considered as significant.

5. APPROVAL OF THE STUDY START

The study will start when:

1) The estimation cost and final protocol is approved and signed (on the second page) by the Study Director (Oncodesign) and an authorised person from Cell Constraint Cancer.
2) The official order is received by Oncodesign from Cell Constraint Cancer.

Any modification after the start of the study will be justified by an amendment to the protocol.

6. DURATION OF THE STUDY

The duration of this study will be 3 to 4 months from the study start and reception of the test substance to the transmission of the final study results and raw data.

7. ARCHIVE

Final protocol and raw data will be kept by Oncodesign for 5 years after the end of the study.

8. CONFIDENTIAL DISCLOSURE AGREEMENT

Cell Constraint Cancer shall have the exclusive ownership of the results of the study and research subject to the present agreement.

Oncodesign agrees to maintain in confidence all confidential information.

It is forbidden for Oncodesign to publish or to communicate any results without the prior written agreement of Cell Constraint Cancer. Cell Constraint Cancer shall freely use the results of the study, for example for public presentations, publications, documentation, marketing authorizations, information for physicians and pharmacists etc.

9. BIBLIOGRAPHY

1. Principe d'éthique de l'expérimentation animale, Directive n°86/609 CEE du 24 Nov. 1986, Décrêt n°87/848 du 19 Oct. 1987, Arrêté d'Application du 19 Avril 1988.
2. United Kingdom co-coordinating committee on cancer research guidelines for welfare of animals in experimental neoplasia, Br. J. Cancer 2010, 102: 1555-1577.
3. CAILLEAU R. et al., J. Natl. Cancer Inst. 1974, 53 : 661-674.
4. SIMPSON-HERREN *et al.*, Cancer Chemother. Rep., 54:143-174, 1970.
5. BISSERY M.C. *et al.*, Bull. Cancer, 78:587, 1991.

PROJECT TIMING

N°		Nom de la tâche
1		**(120005ET100)_CELL CONSTRAINT CANCER**
2		
3		**(120005ET100)_Administrative part of the study**
7		
8		
9		**(120005ET100) ACT_Antitumor activity study of nanopaticles in balb/c nude mice bearing SC MDA-MB-231 tumors**
10		(120005ET100) ACT MDA_amplif cells - A COMMANDER !!
11		(120005ET100) ACT MDA_rcp 45 FE balb/c nude mice - A COMMANDER !!!
12		(120005ET100) ACT MDA_Irr of 45 balb/c nude FE
13		(120005ET100) ACT MDA_Prep of cells
14		(120005ET100) ACT MDA_SC inj cells (45 mice) sur les 2 flancs
15		(120005ET100) ACT MDA_SC inj cells (45 mice) sur les 2 flancs
16		(120005ET100) ACT MDA_visite client pose des aimants
17		(120005ET100) ACT MDA_Rando
18		**(120005ET100) ACT MDA_ BW + TV 44 mice**
43		**(120005ET100) ACT MDA_magnetisation of mice (Q1Dx21 pendant 2 hours, 24 mice)**
65		**(120005ET100) ACT MDA_magnetisation of mice (Q1Dx21 pendant 2 hours, 24 mice)**

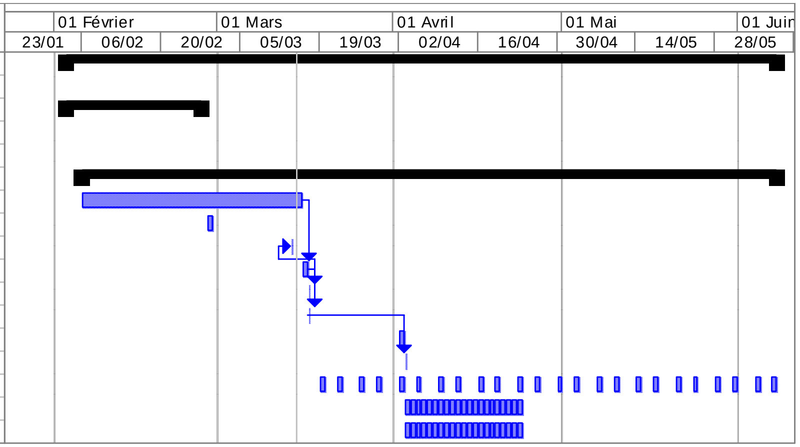

Supplement: S2 Appendix — (PDF) [file pone.0152885.s002.pdf]
