# Supplementary figures and images for: Mechanical Signals Inhibit Growth of a Grafted Tumor In Vivo: Proof of Concept
Source: PLoS One. 2016 Apr 21;11(4):e0152885. doi: 10.1371/journal.pone.0152885 (PMC4839666; doi:10.1371/journal.pone.0152885)

### S3 Appendix: Link between mice number and tumor number

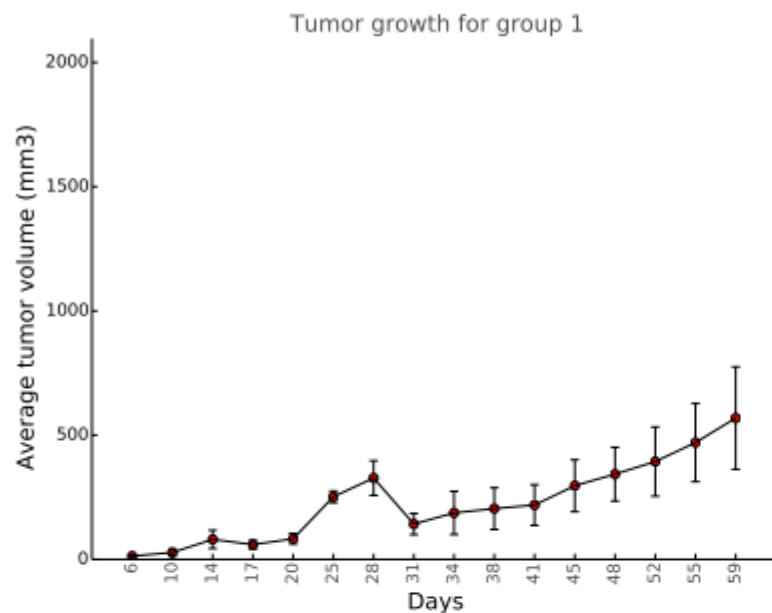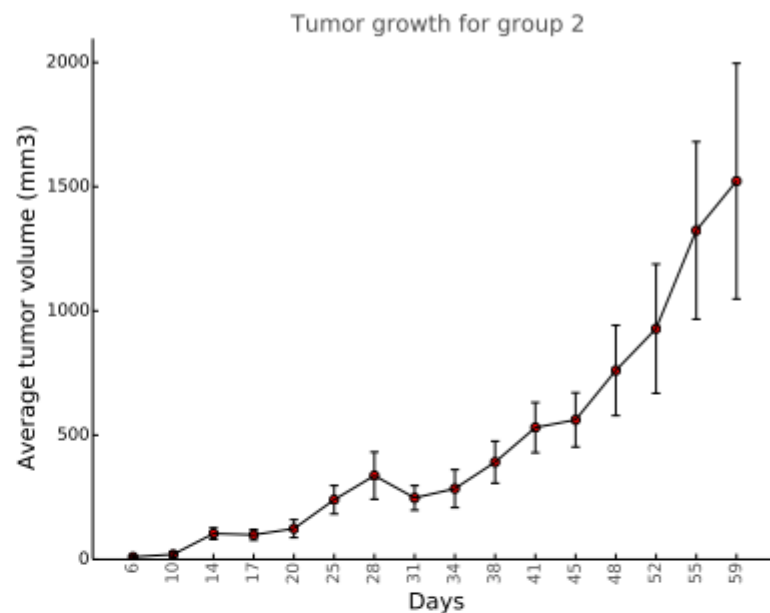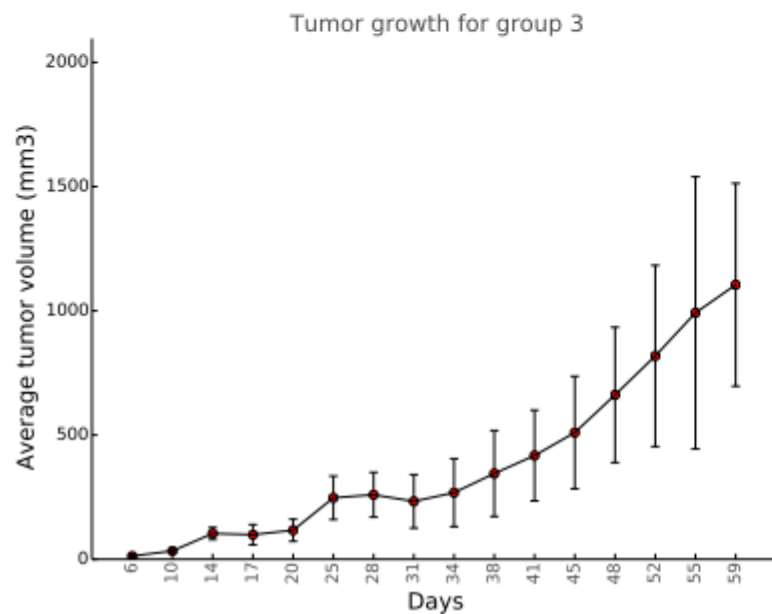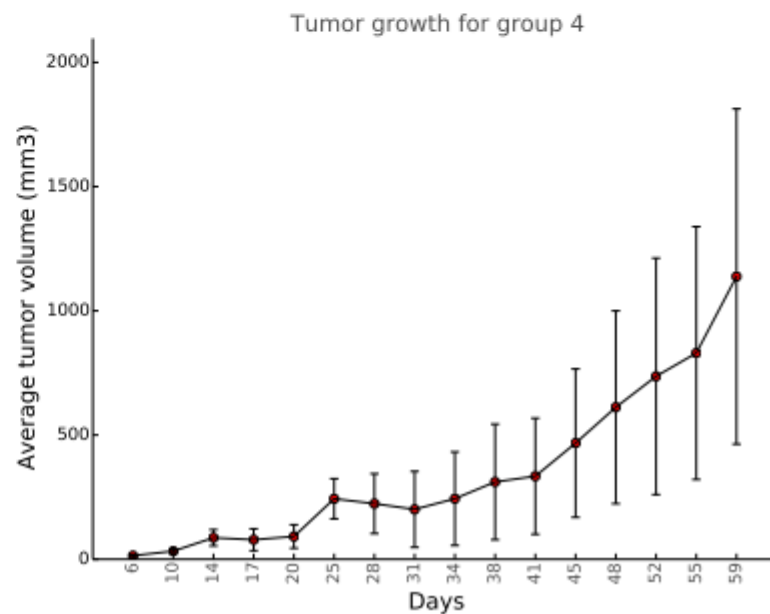

Supplement: S3 Appendix — (PDF) [file pone.0152885.s003.pdf]
