# Supplementary material for: Mechanical Signals Inhibit Growth of a Grafted Tumor In Vivo: Proof of Concept
Source: PLoS One. 2016 Apr 21;11(4):e0152885. doi: 10.1371/journal.pone.0152885 (PMC4839666; doi:10.1371/journal.pone.0152885)
Supplement: S4 Appendix — (PDF) [file pone.0152885.s004.pdf]

S4 Appendix: Statistical report

This document is confidential - Any not authorized reproduction is strictly forbidden.

6 et 8 rue Bellecombe - 69006 LYON
Tél. +33 (0) 472 838 670 - Fax : +33 (0) 472 838 671
http://www.soladis.fr - e-mail : contact@soladis.fr

SARL au capital social de 40 000 € - RCS : 432 515 914
Siret : 432 515 914 00062 – APE : 7022 Z
TVA Intracommunautaire : FR94432515914

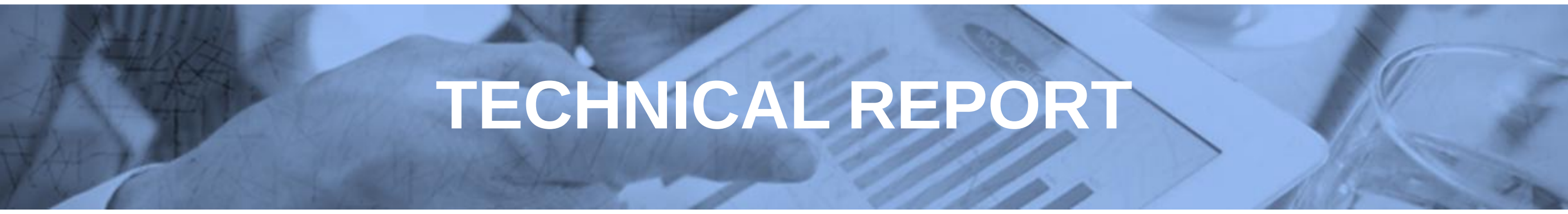

CELL CONSTRAINT & CANCER

Edition A

Recipient

Company	Contact
Cell Constraint & Cancer	M. Rémy BROSSEL

Transmitter

Company	Technical contact	Commercial contact	Administrative contact
SOLADIS – Lyon<br>Direction des opérations	Maxime JURAMY<br>04 72 83 08 24	François CONESA<br>04 72 83 86 72	Dominique BAUD<br>04 72 83 86 70

Document version

Version number	Date	Edition/Modification	Author
**Ed A**	20/05/2014	Initial version	Paul-Edouard BOUVET

Document approval

Version number	Approver's name – SOLADIS	Date	Visa
**Ed A**	Maxime JURAMY	22/05/2014	

This document is confidential - Any not authorized reproduction is strictly forbidden.

Preface

This document is certified as follow:

Historical of version

Version number	Edition/Modification	Date
**Ed A**	Initial version	20/05/2014

Version of author

Version number	Author's name – SOLADIS	Date	Visa
**Ed A**	Paul-Edouard BOUVET	20/05/2014	

Version of approval

Version number	Approver's name – SOLADIS	Date	Visa
**Ed A**	Maxime JURAMY	22/05/2014	

This document is confidential - Any not authorized reproduction is strictly forbidden.

Summary

1. OVERVIEW **5**

2. METHODOLOGY **5**

3. DESCRIPTIVE ANALYSES **6**

3.1 TUMOR VOLUME 6

3.2 SKIN SURFACE 7

3.2.1 ON ALL MICE 7

3.2.2 ON MICE HAVING SURVIVED AFTER 60 DAYS 8

3.3 MUSCLE SURFACE 9

3.3.1 ON ALL MICE 9

3.3.2 ON MICE HAVING SURVIVED AFTER 60 DAYS 10

4. EFFECT OF TREATMENT **11**

4.1 TUMOR VOLUME 11

4.2 SKIN SURFACE 11

4.2.1 ON ALL MICE 11

4.2.1 ON MICE HAVING SURVIVED AFTER 60 DAYS 12

4.3 MUSCLE SURFACE 12

4.3.1 ON ALL MICE 12

4.3.2 ON MICE HAVING SURVIVED AFTER 60 DAYS 13

5. CONCLUSION **14**

6. APPENDIX **15**

6.1 RAW DATA FOR TUMOR VOLUME 15

6.2 RAW DATA FOR SKIN AND MUSCLE SURFACE 15

This document is confidential - Any not authorized reproduction is strictly forbidden.

1. OVERVIEW

As part of a proof of concept, a statistical analysis of the tumor volumes measurements performed on mice is required. This analysis aims at comparing a *Treated* group to three *Control* groups:

- G1 (Treated group): Field + Particles (C/P)
- G2: No Field / Particles (?c/P)
- G3: Field / No Particles (C/?p)
- G4: No Field / No Particles (?c/?p)

These groups will be compared in terms of:

- Tumour volume
- Skin surface
- Muscle surface

2. METHODOLOGY

1. **Import and Data Management**: First the databases will be imported and managed to obtain and format the essential data
2. **Descriptive statistics**: The three variables of interest will be described, in order to check the distribution of the data and the potential presence of outliers.
    - Table of descriptive statistics (n, mean, std, min, max, median, Q1, Q3 and 95% Confidence Interval)
    - Box-plots by groups for each surface

3. **Analysis (Part 1):** Comparison of the treated group (G1) to the three control groups (G2, G3 and G4) for all mice in terms of *Tumour volume*. A one-way ANOVA (Group as unique factor, Tumour volume as response) will be performed. The tumour volume value will be taken at 59 days for mice having survived until this time and at the last measured time for the others.
4. **Analysis (Part 2):** Comparison of the treated group (G1) to the three control groups (G2, G3 and G4) in terms of *Skin Surface* and *Muscle Surface*. Two one-way ANOVAs (Group as unique factor, Surfaces as responses) will be performed. This will be done for all mice and then for mice having survived after 60 days.

For points 3 and 4: if a *group* effect is highlighted, comparisons between groups will be performed.

- Pairwise comparisons between groups G2, G3 and G4 will allow us to understand if there is a significant difference between the control groups.
- If so, G1 will be compared to G2, G3 and G4 separately (*Dunnett test*)
- If not, G1 will be compared to the three groups mixed up (*unadjusted t-test*)

The assumptions of the ANOVA will be checked:

- **Normality of residuals**. If it is not valid, the same model will be applied to log-transformed data. If it is still invalid, a nonparametric test will be performed: A *Kruskal-Wallis* test will allow to test the global *Group* effect and then, if it is significant, Wilcoxon tests will compare groups between them.
- **Homogeneity of variances**. If variances are not homogeneous across groups, this will be taken into account in the model.

This document is confidential - Any not authorized reproduction is strictly forbidden.

3. DESCRIPTIVE ANALYSES

82 identifications of tumors are present in the raw data. Similar identifications are presented below:

3889	4973
3833	4951
3835	2843
3845	4959
3895	2841
4957	3841

79 different tumors have been splitting in groups.

3.1 Tumor volume

Variable	Group	N	Mean (±std)	Median (Q1;Q3)	(Min;Max)	Confidence limits (95%)
Tumor volume	1	18	382.862 (±281.666)	246.010 (159.160;529.430)	(94.240;965.500)	[242.793;522.932]
	2	21	1085.634 (±756.456)	1334.170 (333.760;1784.130)	(95.500;2105.870)	[741.300;1429.969]
	3	8	564.826 (±394.929)	595.840 (161.255;914.295)	(86.770;1089.060)	[234.657;894.995]
	4	32	635.110 (±612.701)	387.640 (174.770;928.480)	(69.650;1996.430)	[414.207;856.012]

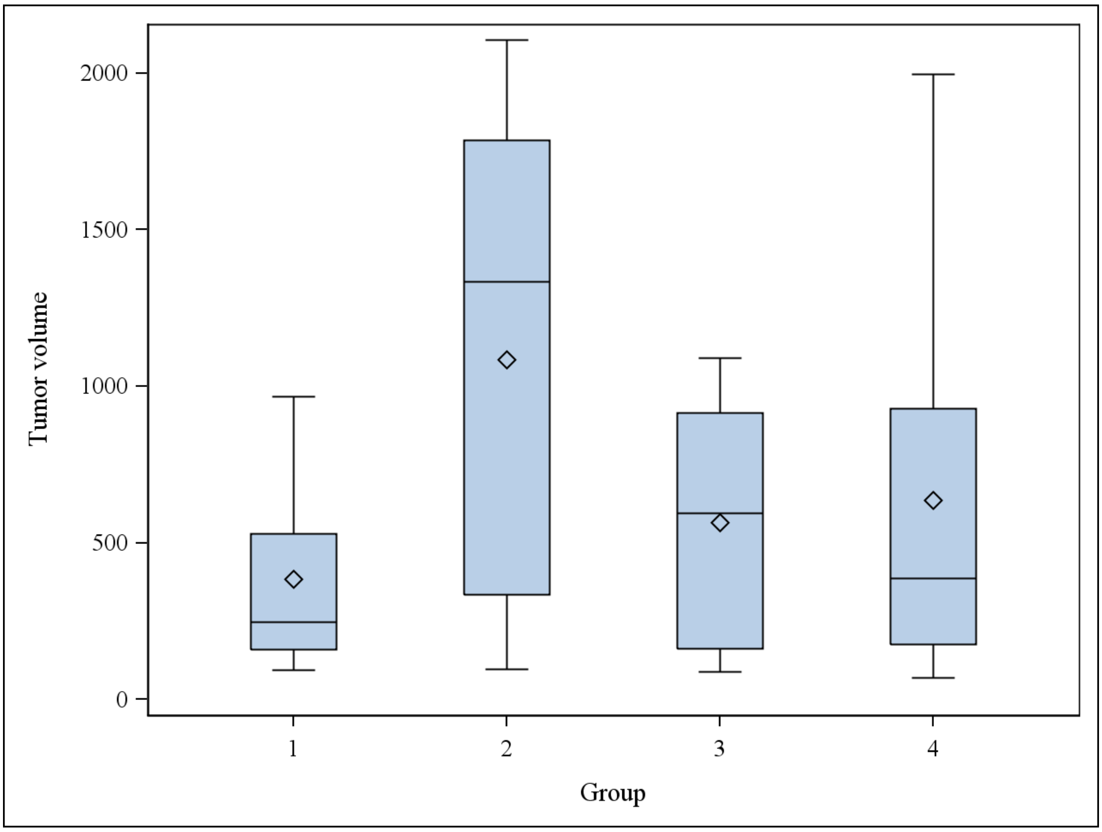

This document is confidential - Any not authorized reproduction is strictly forbidden.

3.2 Skin surface

The skin surface was not measured on all the tumors (insufficient quality of the cut). Total surface measurements are available for 51 tumors.

3.2.1 On all mice

Variable	Group	N	Mean (±std)	Median (Q1;Q3)	(Min;Max)	Confidence limits (95%)
Skin surface	1	9	10.937 (±12.764)	5.582 (4.203;9.776)	(3.023;42.409)	[1.126;20.748]
	2	14	20.594 (±8.661)	20.795 (16.313;26.412)	(4.919;34.330)	[15.593;25.595]
	3	4	17.320 (±4.646)	16.456 (13.591;21.049)	(13.215;23.152)	[9.927;24.712]
	4	19	17.084 (±12.522)	14.784 (8.965;21.044)	(1.843;55.475)	[11.049;23.120]

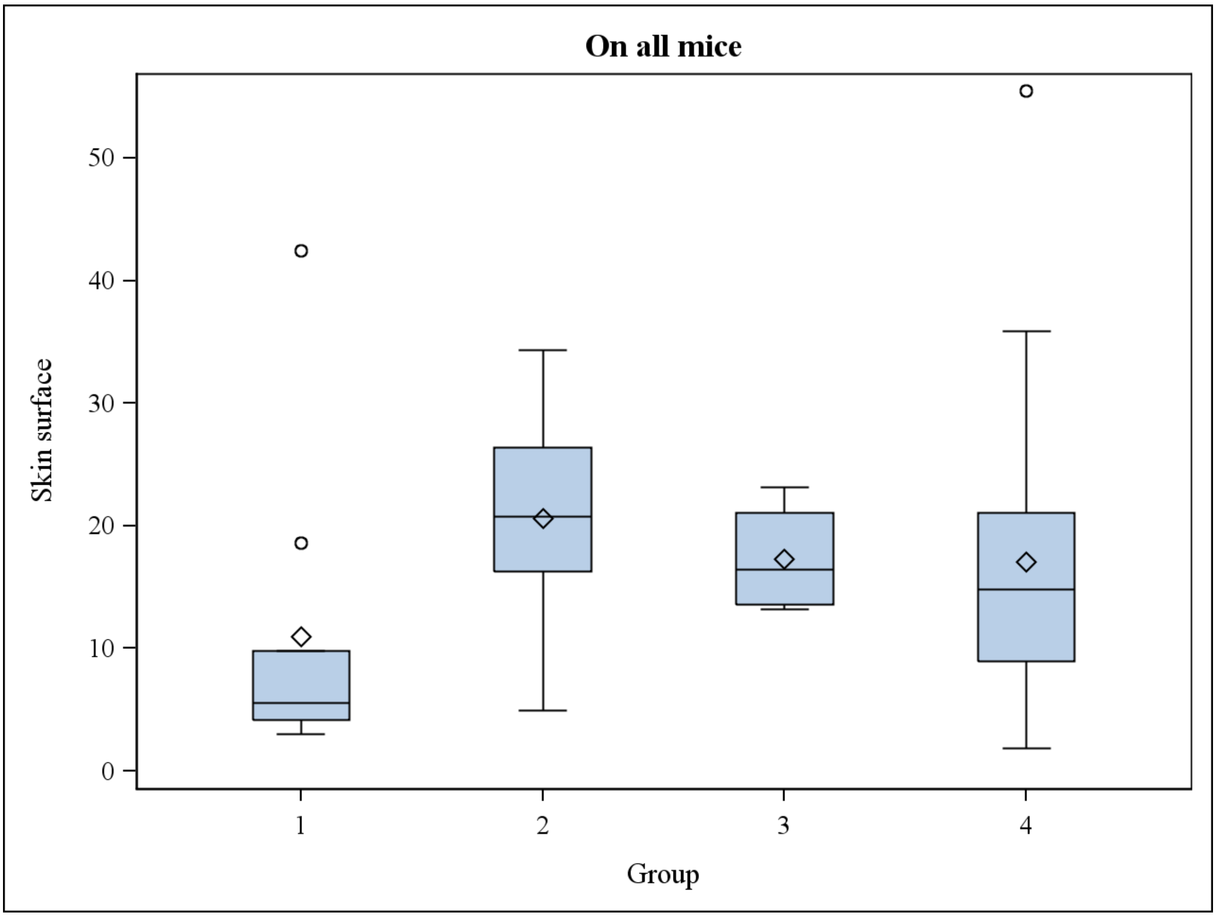

This document is confidential - Any not authorized reproduction is strictly forbidden.

3.2.2 On mice having survived after 60 days

Variable	Group	N	Mean (±std)	Median (Q1;Q3)	(Min;Max)	Confidence limits (95%)
Skin surface	1	4	19.097 (±16.464)	14.199 (7.679;30.515)	(5.582;42.409)	[-7.101;45.295]
	2	10	24.575 (±6.077)	23.836 (19.746;28.830)	(16.313;34.330)	[20.227;28.923]
	3	4	17.320 (±4.646)	16.456 (13.591;21.049)	(13.215;23.152)	[9.927;24.712]
	4	10	25.121 (±12.246)	20.540 (18.080;27.254)	(14.784;55.475)	[16.361;33.881]

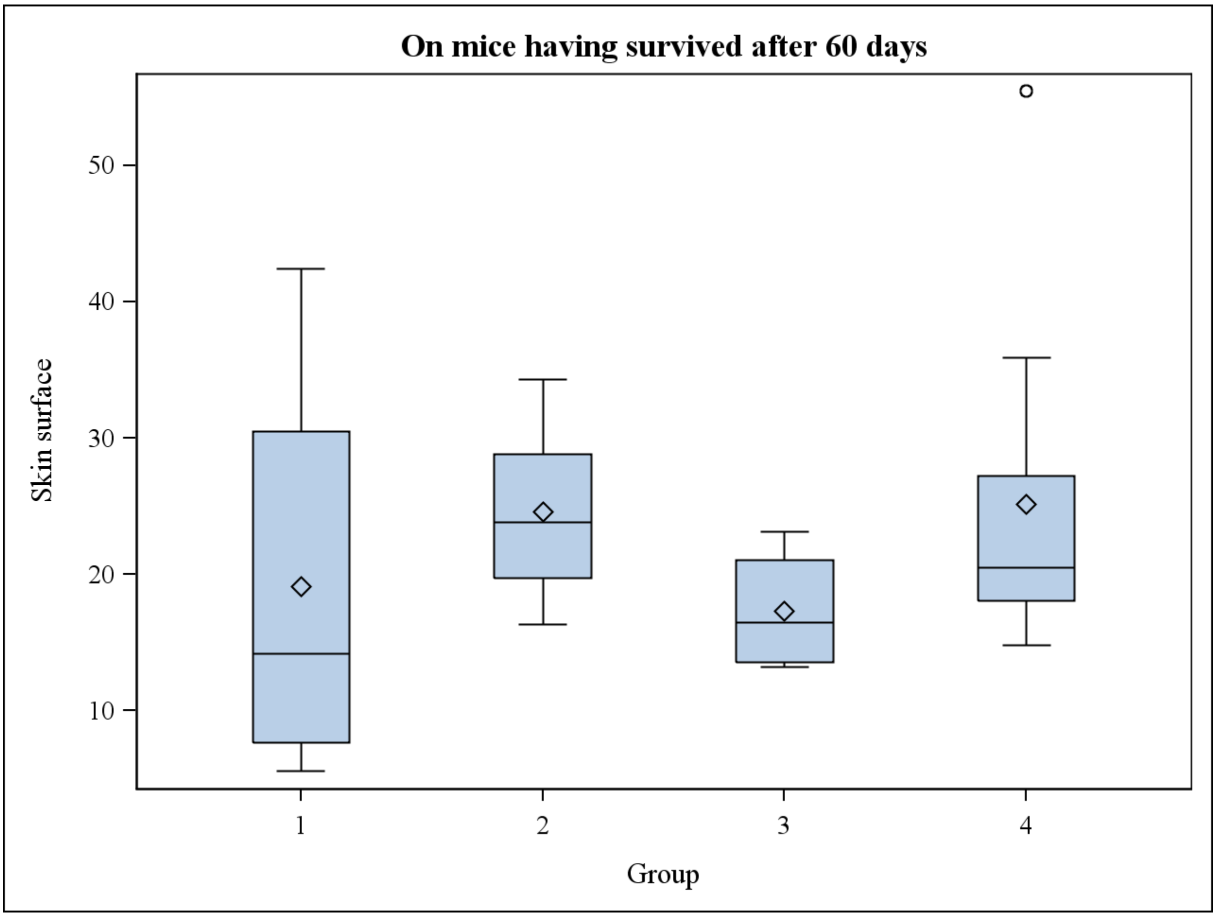

3.3 Muscle surface

The muscle surface was not measured on all the tumors (insufficient quality of the cut). Total surface measurements are available for 51 tumors.

3.3.1 On all mice

Variable	Group	N	Mean (±std)	Median (Q1;Q3)	(Min;Max)	Confidence limits (95%)
Muscle surface	1	9	7.727 (±8.390)	4.714 (3.618;7.211)	(1.923;29.214)	[1.278;14.177]
	2	14	23.108 (±14.047)	24.993 (11.744;28.378)	(5.225;55.012)	[14.997;31.218]
	3	4	21.431 (±9.419)	17.952 (15.540;27.322)	(14.578;35.242)	[6.444;36.418]
	4	19	18.650 (±14.923)	12.521 (6.037;28.605)	(1.531;51.792)	[11.457;25.842]

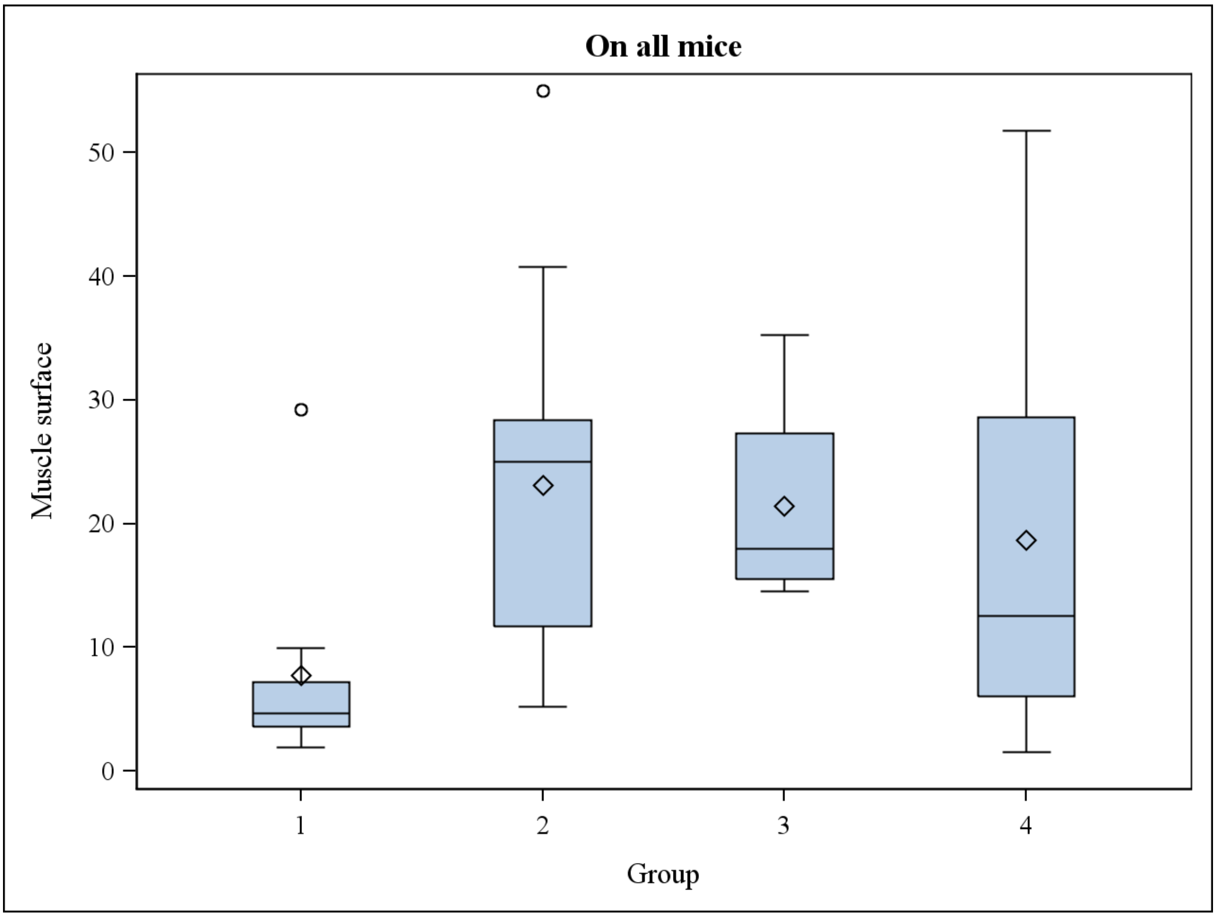

3.3.2 On mice having survived after 60 days

Variable	Group	N	Mean (±std)	Median (Q1;Q3)	(Min;Max)	Confidence limits (95%)
Muscle surface	1	4	12.761 (±11.184)	8.597 (5.923;19.599)	(4.634;29.214)	[-5.036;30.557]
	2	10	29.114 (±11.898)	26.074 (24.813;33.533)	(11.744;55.012)	[20.603;37.626]
	3	4	21.431 (±9.419)	17.952 (15.540;27.322)	(14.578;35.242)	[6.444;36.418]
	4	10	28.589 (±13.956)	28.075 (12.521;40.303)	(10.948;51.792)	[18.606;38.573]

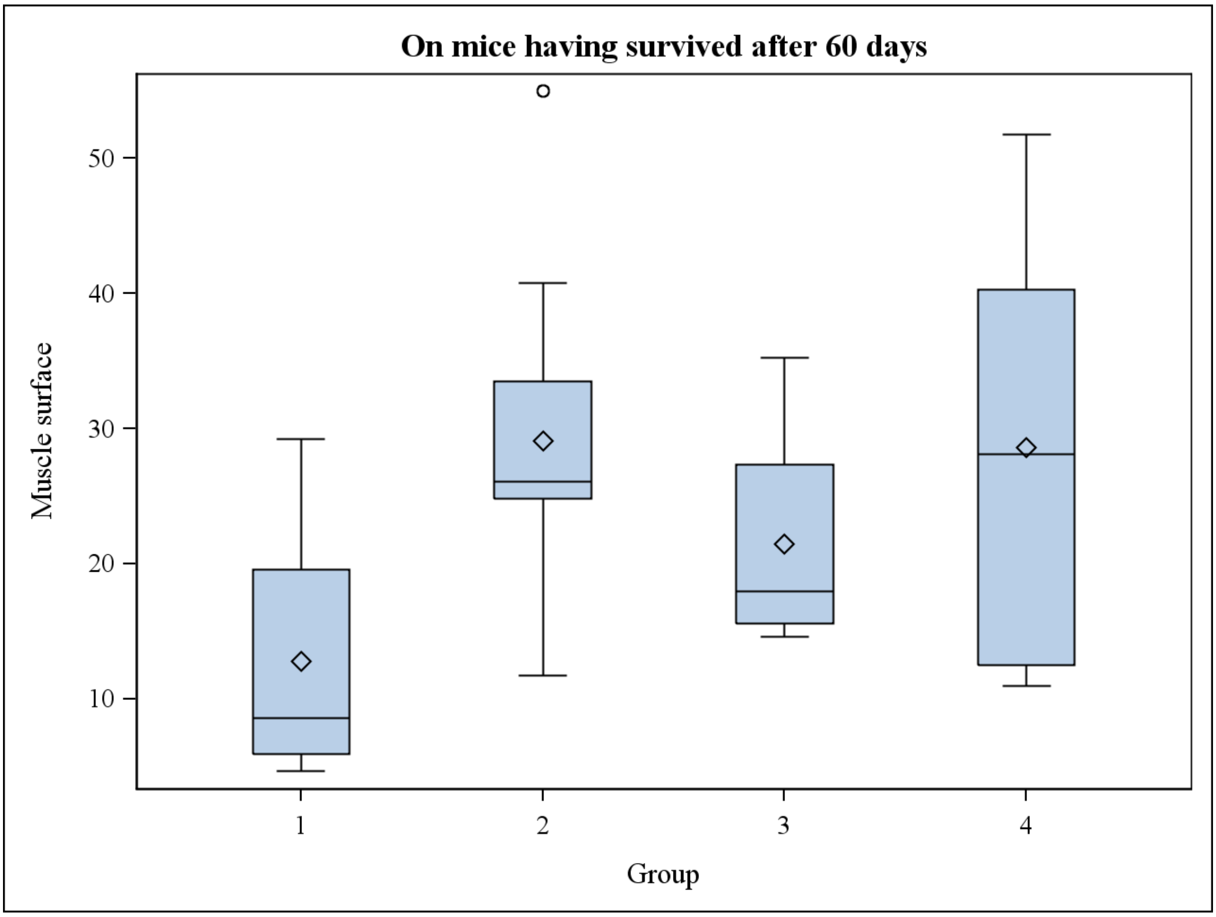

This document is confidential - Any not authorized reproduction is strictly forbidden.

4. EFFECT OF TREATMENT

4.1 Tumor volume

Normality of residuals is not verified with raw data (p<0.01) and also with data transformed in log (p<0.01). So a nonparametric test is performed.

The pairwise comparisons between groups G2, G3 and G4 are performed with a Wilcoxon test using a Bonferroni-Holm adjustment.

Comparison	Signification (Bonferroni-Holm adjustment)
G2 vs G3	Not Significant (p=0.359)
G2 vs G4	Not Significant (p=0.114)
G3 vs G4	Not Significant (p=0.947)

All p-values are higher than 0.05, so there is no significant difference between untreated groups. The three groups are mixed up. A Wilcoxon test is performed between Treated (G1) and Untreated (G2, G3 and G4).

Comparison	Signification (Wilcoxon test)
Treated (G1) vs. untreated (G2,G3,G4)	Not Significant (p=0.067)

There is no significant difference between G1 and other groups (p=0.067).

4.2 Skin surface

4.2.1 On all mice

Normality of residuals is not verified with raw data (p=0.0002) but is verified with data log transformed (p=0.611) so a one-way ANOVA is performed with log-transformation.

Skin surface - on all mice - Global Effect	
**Effect**	**Signification (pvalue)**
Group	Significant (p=0.021)

The first result shows that groups have an effect on skin surface.
Next table presents pairwise comparisons using Tukey's adjustment between untreated groups (one-way ANOVA):

Skin surface - on all mice - Comparison untreated groups		
**Comparisons**	**Estimate**	**Signification (pvalue) (Tukey's adjustment)**
G2 vs. G3	0.037	Not Significant (p=0.973)
G2 vs. G4	0.141	Not Significant (p=0.372)
G3 vs. G4	0.104	Not Significant (p=0.799)

This document is confidential - Any not authorized reproduction is strictly forbidden.

All p-values are higher than 0.05, so there is no significant difference between the three groups untreated. A mixed up of this 3 groups is realized.

Finally, one-way ANOVA is performed using contrast option:

Skin surface - on all mice - Final comparison			
**Comparison**	**Estimate of differences**	**Signification (pvalue)**	**95% IC**
G1 vs (1/3*G2 + 1/3*G3 + 1/3*G4)	-0.344	Significant (p=0.005)	[-0.576;-0.112]

Skin surface on treated group is significantly different to untreated groups (mixed up). Skin surface on group treated is lower than on group untreated (estimate of difference = -0.344 log).

4.2.1 On mice having survived after 60 days

Normality of residuals is not verified with raw data (p=0.002) but is verified with data log transformed (p=0.2086) so a one-way ANOVA is performed with log-transformation.
Variability of group 1 is not homogenous with variabilities of the 3 others groups. So, this heterogeneity was taken into account in the model with the option “group=”.

Skin surface - on mice having survived after 60 days	
**Effect**	**Signification (pvalue)**
Group	Not Significant (p=0.172)

On one-way anova, group effect is not significant (p=0.172). There is no significantly difference between the 4 groups on mice having survived after 60 days.

4.3 Muscle surface

4.3.1 On all mice

Normality of residuals is not verified with raw data (p=0.0063) but is verified with data log transformed (p=0.937) so a one-way ANOVA is performed with log-transformation.

Muscle surface - on all mice - Global Effect	
**Effect**	**Signification (pvalue)**
Group	Significant (p=0.004)

The first result shows that groups have an effect on muscle surface.

This document is confidential - Any not authorized reproduction is strictly forbidden.

Next table presents pairwise comparisons using Tukey's adjustment between untreated groups (one-way ANOVA):

Muscle surface - on all mice - Comparison untreated groups		
**Comparisons**	**Estimate**	**Signification (pvalue) (Tukey's adjustment)**
G2 vs. G3	-0.028	Not Significant (p=0.988)
G2 vs. G4	0.159	Not Significant (p=0.377)
G3 vs. G4	0.187	Not Significant (p=0.571)

All p-values are higher than 0.05, so there is no significant difference between the three groups untreated. A mixed up of this 3 groups is realized.

Finally, a one-way ANOVA is performed using contrast option:

Muscle surface - on all mice - Final comparison			
**Comparison**	**Estimate of differences**	**Signification (pvalue)**	**95% IC**
G1 vs (1/3*G2 + 1/3*G3 + 1/3*G4)	-0.485	Significant (p=0.001)	[-0.749;-0.222]

Muscle surface on treated group is significantly different to untreated groups (mixed up). Muscle surface on group treated is lower than on group untreated (Estimate of difference=-0.485 log).

4.3.2 On mice having survived after 60 days

Normality of residuals is verified with raw data (p=0,10) so a one-way ANOVA is performed.

Muscle surface - on mice having survived after 60 days	
**Effect**	**Signification**
Group	Not Significant (p=0.093)

On one-way ANOVA, group effect is not significant (p=0.093). There is no significantly difference of muscle surface between G1 and other groups on mice having survived after 60 days.

This document is confidential - Any not authorized reproduction is strictly forbidden.

5. CONCLUSION

Variable	Population	Model	Group effect	Comparison of G1 vs G2/G3/G4
**Tumor volume**	All mice	Nonparametric	0.067 (NS)	/
**Skin surface**	All mice	Parametric (logarithmic transformation)	0.021 (S)	0.005 (S)
	Mice having survived after 60 days	Parametric (logarithmic transformation)	0.172 (NS)	/
**Muscle surface**	All mice	Parametric (logarithmic transformation)	0.004 (S)	0.001 (S)
	Mice having survived after 60 days	Parametric	0.093 (NS)	/

Significance:

- NS : Not significant
- S : Significant

This document is confidential - Any not authorized reproduction is strictly forbidden.

6. APPENDIX

6.1 Raw data for tumor volume

Données_Excel.xls

6.2 Raw data for skin and muscle surface

Données_SQL.xls

This document is confidential - Any not authorized reproduction is strictly forbidden.
